# Hidradenitis suppurativa patients exhibit a distinctive and highly individualized skin virome

**DOI:** 10.1128/msystems.01290-25

**Published:** 2025-12-15

**Authors:** Daan Jansen, Lene Bens, Jeroen Wagemans, Sabrina I. Green, Tom Hillary, Tine Vanhoutvin, An Van Laethem, Séverine Vermeire, João Sabino, Rob Lavigne, Jelle Matthijnssens

**Affiliations:** 1 Department of Microbiology, Immunology and Transplantation, Laboratory of Clinical and Epidemiologucal Virology, KU Leuven, Rega Institute 26657, Leuven, Belgium; 2 Department of Biosystems, KU Leuven 26657, Leuven, Belgium; 3 Department of Dermatology, KU Leuven University Hospitals Leuven, Leuven, Belgium; 4 Department of Gastroenterology and Hepatology and Department of Chronic Diseases & Metabolism KU Leuven, University Hospital Leuven, Leuven, Belgium; Institute of Biochemistry and Biophysics of the Polish Academy of Sciences, Warsaw, Poland

**Keywords:** hidradenitis suppurativa, virome, microbiome, bacteriophages, metagenomics

## Abstract

Hidradenitis suppurativa (HS) is a chronic inflammatory disease characterized by recurring skin lesions. Despite ongoing research, the exact cause underlying initiation and progression of disease remains unknown. While prior research has linked the skin microbiota to HS pathology, the role of viruses has remained unexplored. To investigate the skin virome, metagenomic sequencing of viral particles was performed on 144 skin samples from 57 individuals (39 HS patients and 18 controls). It was found that the virome is not only linked to BMI, but also to the presence and severity of HS, marking a diverging viral profile in the progression of disease. Despite no differences in alpha-diversity, HS patients exhibited a significantly higher beta-diversity compared to healthy controls, indicating a more personalized virome with reduced viral sharing among patients. We identified distinct groups of commonly shared phages, referred to as the core phageome, associated with either healthy controls or patients. Healthy controls displayed a higher abundance of two core *Caudoviricetes* phages predicted to infect *Corynebacterium* and *Staphylococcus*, comprising normal skin commensals. In contrast, HS patients carried previously uncharacterized phages that were more prevalent in advanced stages of the disease, which likely infect *Peptoniphilus* and *Finegoldia*, known HS-associated pathogens. Interestingly, genes involved in superinfection exclusion and antibiotic resistance could be found in phage genomes of healthy controls and HS patients, respectively. In conclusion, we report the existence of distinct core phages that may have clinical relevance in HS pathology by influencing skin bacteria through mechanisms such as superinfection exclusion and antibiotic resistance.

**IMPORTANCE:**

An increasing body of research showed that the microbiome has an important role in complex human disease. In line with this, here, we analyzed a longitudinal HS cohort and found a relationship between the skin virome and HS pathology. This relationship was defined by distinct groups of phages associated with either healthy controls or HS patients, yet, in both instances, capable of enhancing bacterial fitness. In healthy individuals, these phages were widely shared, fostering symbiosis by ensuring stability of the commensal skin microbiota. Conversely, in HS patients, these phages revealed a more individualistic nature and could contribute to dysbiosis by providing antibiotic resistance genes to bacterial pathogens. Overall, these findings point to a potential clinical significance of the virome in understanding and addressing HS pathology.

## INTRODUCTION

Hidradenitis suppurativa (HS) is a chronic inflammatory skin disease of unknown origin that usually develops in skin folds, such as the inguinal and axillary regions (1). The disease is systemic in nature and is frequently associated with comorbidities, including but not limited to metabolic syndrome, spondylarthritis, and inflammatory bowel disease (IBD) (2–4). The etiology of HS is complex and involves a combination of factors including the environment, lifestyle, hormone imbalances, genetics, and the microbiota (5–9). The interplay between these factors can cause the initiation and development of inflammation around the hair follicles, leading to follicular plugging, abscess formation, tissue damage, and ultimately, scar formation (8, 10).

Of these factors, the microbiota is of particular interest due to its intricate and enigmatic role in the initiation and development of HS (11). The human microbiota is known as a complex ecosystem of microorganisms, including viruses, fungi, bacteria, archaea, and protozoa, that inhabit the skin and various other mucosal environments. These microorganisms establish a commensal relationship with the human host and provide benefits, such as protecting against invading pathogens. However, this delicate balance is sometimes disrupted, leading to a state called dysbiosis. Dysbiosis has been linked to numerous diseases, including IBD, asthma, depression (12–14), and various skin conditions, such as psoriasis, atopic dermatitis, and acne vulgaris (15–17).

In this context, a growing body of evidence has linked HS to changes in the bacterial component of the skin microbiota (18–22). These changes are found in various skin regions, including dry, moist, and sebaceous areas (23). HS is a skin condition that commonly affects the skin folds surrounding moist skin regions and is typically characterized by a higher proportion of anaerobic bacteria, such as *Peptoniphilus*, *Porphyromonas*, *Prevotella*, *Finegoldia,* and several other microbes (18–20). In comparison, healthy individuals tend to have a high proportion of aerobic bacteria that are naturally found in the skin as commensals, including *Corynebacterium, Cutibacterium,* and *Staphylococcus* (18, 20–23). HS is not linked to a single bacterium, but rather to a mixture of bacteria that are associated with the development and progression of the disease. In that regard, there have been reports of changes in diversity, but these findings are not always consistent (18–20). While some studies indicate no differences (19), others suggest a higher bacterial alpha diversity on the skin of patients seemingly associated with increasing severity of disease (18, 20).

While bacterial skin dysbiosis is a frequently described event in the pathophysiology of HS, the skin virome (the collection of viruses infecting human cells, as well as bacteriophages infecting resident bacteria) remains largely unstudied (24). The skin virome could nonetheless play an important role in disease, as bacteriophages can control bacterial populations through both lytic and lysogenic infection (25–27). While lytic infection leads to the lysis of bacterial cells and reduces bacterial numbers, lysogenic conversion (the integration of the phage genome in the bacterial genome) allows for the transfer of new genes into the bacterial genome that can influence its metabolism, virulence, and antibiotic resistance (28–31). These mechanisms have been shown to influence bacterial communities in various inflammatory skin conditions but have not yet been described in HS (32, 33). For example, in atopic dermatitis, phage SaGU1 selectively infects *Staphylococcus aureus* and suppresses its overgrowth without affecting beneficial *Staphylococcus epidermidis* (32). In acne vulgaris, decreased abundance of *Cutibacterium acnes* phages in lesional skin correlated with increased bacterial proliferation and disease severity (33). These examples illustrate how phage-bacteria dynamics can shape the skin microbiome and contribute to disease outcomes*,* thereby underscoring the importance of investigating such interactions in HS. In addition, eukaryotic viruses can directly infect the human skin, activate the immune system, and contribute to disease, as demonstrated by associations between papillomaviruses, polyomaviruses, and herpesviruses with various dermatological disorders (34–37).

In the present study, we aim to investigate the potential role of the skin virome (both eukaryotic and prokaryotic viruses) in HS patients. To achieve this, we conducted an in-depth analysis of skin swabs (*n =* 144) collected from a longitudinal comparative study consisting of 39 patients and 18 healthy controls. By examining these samples, we identified a distinct viral signature that enriches our understanding of the role of the skin microbiome as a whole in the pathophysiology of HS.

## MATERIALS AND METHODS

### Study design and sample collection

The longitudinal comparative study consisted of 18 healthy individuals and 39 HS patients. Healthy controls were included based on the absence of any dermatological illness and were carefully matched, as closely as possible, for host variables that could potentially impact the microbiota, such as age, sex, ethnicity, and others (Table S1). Skin samples were collected from both patients (lesions) and healthy controls using ESwabs (1 mL Liquid Amies, Copan, Italy). To ensure a comprehensive data set, patients were sampled at various locations on the body where skin was affected, at different time points (ideally at baseline, 6 months, and 12 months). A total of 144 samples were collected, consisting of 108 patient samples and 36 healthy control samples. A more detailed overview of the study design and sample collection was previously described by Bens and colleagues (38).

### Viral metagenomics

To perform viral metagenomics on skin swabs, the NetoVIR protocol was used as described before (39). The skin samples were homogenized using a tabletop vortex, and the complete volume (1 mL) was transferred to a 1.5-mL receiver tube. The homogenate was centrifuged at 17,000 × *g* for 3 min, and the resulting supernatant was passed through a 0.8-µm PES filter (Sartorius). A nuclease treatment was then performed using micrococcal nuclease (New England Biolabs) and benzonase (Novagen) at 37°C for 2 h. Viral nucleic acids were extracted using the QIAMP Viral RNA mini kit (60 µL, Qiagen, Venlo, Netherlands) without the addition of carrier RNA. This extraction kit has been previously shown to extract both RNA and DNA viral genomes without introducing any major bias (40). The Complete Whole Transcriptome Amplification kit (WTA2, Sigma-Aldrich) was employed with minor adaptations (94°C for 2 min, and 17 cycles of 94°C for 30 s and 70°C for 5 min) to perform reverse transcription and random amplification of the extracted nucleic acids. To address concerns regarding the potential amplification bias towards viruses with a circular single-stranded DNA genome induced by multiple displacement amplification (MDA), a number of points need to be emphasized. First, the WTA2 kit only involved a limited MDA step followed by a classical PCR. Second, the NetoVIR protocol was previously benchmarked on a diverse mock-virome encompassing both linear and circular genomes and did not reveal any indication of such amplification bias (39). Next, a purification step was performed on the amplified PCR product using the MSB Spin PCRapace kit (Invitek Molecular), followed by a concentration measurement using the Qubit dsDNA HS Assay Kit. Sequencing libraries were prepared from the purified PCR product using the Nextera XT DNA Library kit (Illumina). After library preparation, the sequencing libraries (25 µL) were purified using Agentcourt AMPure XP beads (15 µL, ratio_beads/dsDNA_ = 0.6, Beckman Coulter) to remove impurities and obtain high-quality libraries. The purified libraries were then assessed for their fragment sizes using the High Sensitivity DNA kit on the Bioanalyzer 2100 (Agilent Technologies). After applying the NetoVIR protocol, a total of 138 high-quality sample libraries were obtained for sequencing. The VIB Nucleomics core performed the sequencing of the libraries using a NovaSeq6000 S1 sequencer (2 × 150 bp, paired end) as the final step. Following the sequencing process, a total of five additional samples were excluded due to a low number of raw reads, indicating low-quality samples, resulting in a total of 133 data sets for further bioinformatic analysis. An elaborate description of the bioinformatic processing, viral identification and classification, diversity analyses, and other methodologies can be found in the Supplemental Methods.

## RESULTS

### The skin virome of a comparative HS cohort is characterized by a high proportion of unclassified phages

Skin swabs (*n =* 144) were collected from patients (*n =* 39) and healthy controls (*n =* 18) in a comparative HS cohort (*n =* 57). Patients were categorized into three groups based on the severity of the disease, namely Hurley stage 1 (*n =* 10), Hurley stage 2 (*n =* 19), and Hurley stage 3 (*n =* 10). To obtain a complete data set, patients were sampled at multiple areas of their body where skin was affected (axillary, abdomen, chest, gluteal, groin, and genitals), at different time intervals (ideally at baseline, six months, and 12 months). Healthy control samples were collected once from the axillary (*n =* 18, 50% of samples) and groin and genitals (*n =* 18, 50% of samples) to match the vast majority of the patients’ samples (*n =* 82, 76.0% of samples; Table S1). Of note, 13 of the included patients were diagnosed with IBD, which enabled a comparison of the skin virome of HS patients with and without IBD (Supplemental Methods; Table S1). Next, the virome was characterized by enriching and sequencing viral genomes using the NetoVIR protocol (39). Bioinformatic analyzes on 4.1 billion paired-end reads (0.62 TB, x̄ = 28.6 million reads per sample) were performed to obtain a high-quality (HQ) viral data set (“HQ Viral NR-Contigs”; Fig. S1). The HQ viral data set revealed a wide range of recovered viruses across samples, encompassing 40.2% of the obtained HQ reads (Supplemental Methods; Fig. S2). This observation is likely attributed to factors, such as incomplete public skin phage databases as well as genome fragmentation, corroborating the findings of Graham and colleagues (31). To include a greater number of partially sequenced phage genomes in our analysis and obtain a more representative picture of the skin virome in the complete HS cohort (Healthy + HS), we employed a clustering approach. This method grouped non-redundant contigs into clusters at a “family-like” similarity level using Markov clustering. All clusters, including one or more HQ phages (Supplemental Methods, Fig. S1), were considered as phage “family-like” viral clusters (FLVC). The FLVC approach increased the retained number of quality-controlled reads to 66.9% (Fig. S2). Regardless of the used viral identification approach (individual HQ-contigs or FLVCs), less than half of the bacteriophage contigs could be taxonomically classified at class level or lower (HQ-contigs_classified_ = 25.5%, FLVC_classified_ = 40.2%; Fig. S3), thereby highlighting the high proportion of previously undescribed phages. Most of the classified skin phage genomes belonged to *Caudoviricetes* (HQ-contigs_classified_ = 19.3%, FLVC_classified_ = 39.1%), with only a small fraction classified as *Malgrandaviricetes* (HQ-contigs_classified_ = 6.2%, FLVC_classified_ = 1.10%).

### A small and diverse eukaryotic virome with anelloviruses confined to patients

The eukaryotic virome was relatively small, with eukaryotic viruses found in up to 51.2% of skin samples (or 66.6% of individuals), representing 15.9% of the quality-controlled viral reads (FLVC approach; Fig. S2). In contrast, phages were far more abundant and accounted for 84.1% of the quality-controlled viral reads (FLVC approach; Fig. S2). The four major eukaryotic viral families (prevalence ≥15%) recovered were *Papillomaviridae*, *Totiviridae*, *Alphaflexiviridae,* and *Anelloviridae* (Fig. S4A). These families typically comprise viruses known to infect humans (*Papillomaviridae*), fungi and protozoa (*Totiviridae*), and plants (*Alphaflexiviridae*), as well as small circular viruses with unknown host (*Anelloviridae*). Despite the frequent occurrence of these viral families, they did not reveal a significantly different relative abundance (RA) between healthy controls and HS patients (*n = 38*, Mann-Whitney U, AdjP ≥ 0.05; Fig. S4B; Table S3). A noteworthy observation from our study was that anelloviruses, for which the host cells and biological function in the human body are still unknown (41), were exclusively detected in skin samples of HS patients. In addition, the detected totiviruses were predicted to infect the fungi *Malassezia*, which typically exist as a commensal on human skin surfaces (42). Another peculiar finding included the detection of members of the *Dicistroviridae* and *Trichomonasvirus* capable of infecting the house dust mite and the protozoan parasite *Trichomonas*, respectively, in several of the patients. Ultimately, our findings suggest that eukaryotic viruses are unlikely to contribute meaningfully to HS pathology, as they were present at low levels and did not vary consistently between healthy and diseased individuals.

### The skin phageome composition is associated with the presence and severity of HS

**Fig 1:**
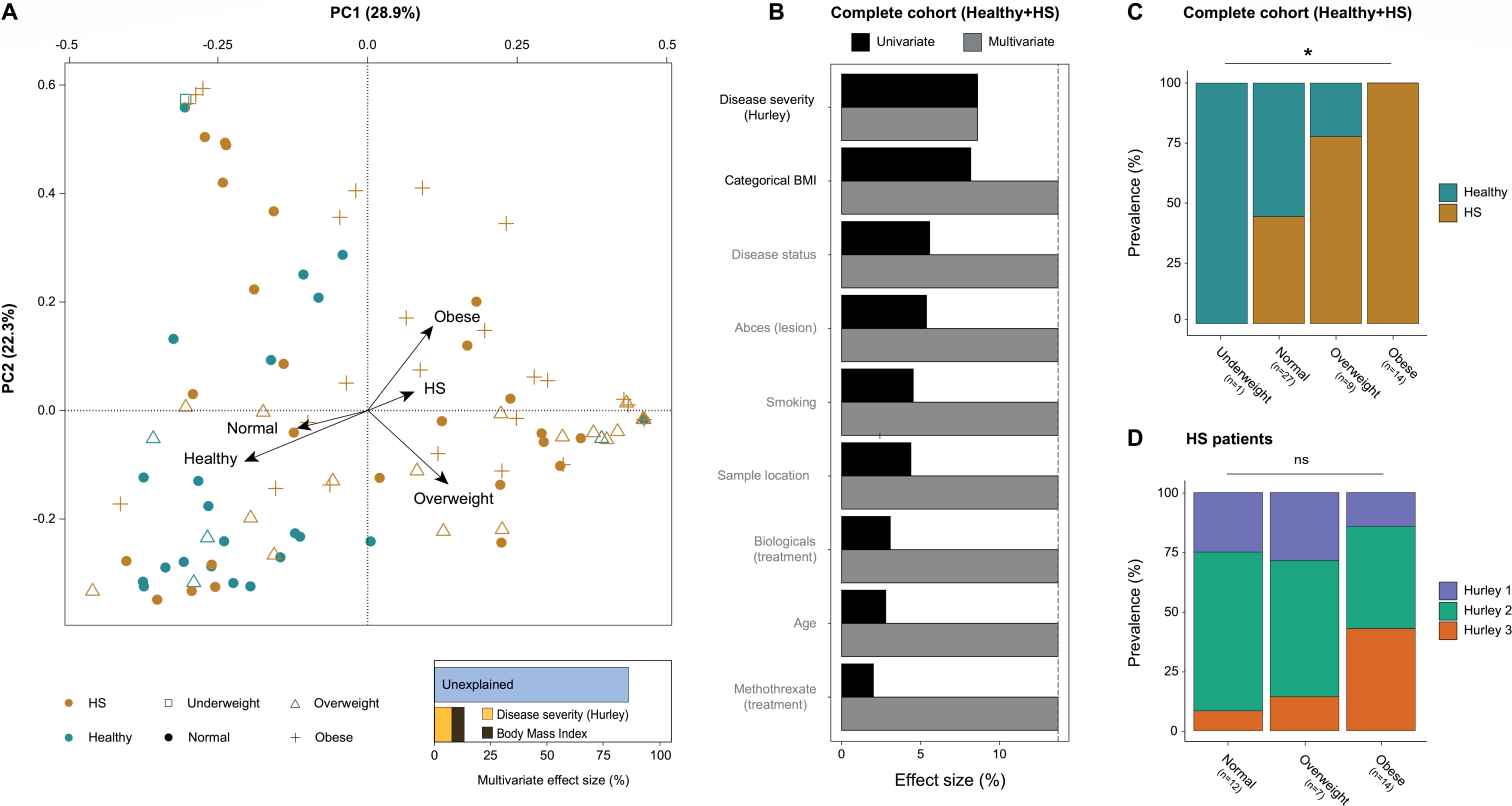
Skin virome variation in the complete study cohort. (**A**) A principal coordinate analysis visualizing the inter-individual differences of the skin virome composition (*n =* 99, FLVC level, sample-level, Bray-Curtis dissimilarity) of the complete cohort (Healthy + HS), colored by disease status and with shapes depicted by categorical BMI. The bar plot at the bottom illustrates the significant covariates of the skin virome (86.3% unexplained); arrows on the plot indicate the effect sizes of these significant covariates (underweight [*n =* 1] not shown). (**B**) Panel shows the metadata variables significantly associated with the skin virome composition in the complete cohort (*n =* 99, dbRDA, FLVC level, sample-level, Bray-Curtis dissimilarity). The effect sizes of covariates are shown independently as univariate analysis (in black) or as a cumulative model (in gray). (**C, D**) Panels illustrate the associations between disease status (*n =* 51*,* patient-level, chi-squared test, AdjP < 0.05) or disease severity (*N =* 33, patient-level, chi-squared test, AdjP ≥ 0.05) and categorical BMI. Multiple testing adjustment (Benjamini-Hochberg method) was performed, and significant associations (AdjP < 0.05) are represented by an asterisk (*). Abbreviations: hidradenitis suppurativa (HS)/Healthy controls (Healthy), complete cohort (Healthy + HS), and non-significant (ns).

To investigate the relationship between HS pathology and the skin phageome, we analyzed the compositional variation of the phageome in the HS cohort. In doing so, a stepwise distance-based redundancy analysis (dbRDA) was used to identify factors associated with the compositional variation of the phageome (Healthy + HS; sample-level, Table S4). First, this analysis revealed significant associations with both disease severity and categorical body mass index (cBMI), supporting a link between HS pathology and skin phages (*n =* 99, sample-level, multivariate dbRDA, FLVC-level, disease severity R_2_ = 8.60%, cBMI R_2_ = 5.10%, AdjP < 0.05; Fig. 1A and B). The fact that healthy controls were not fully matched, and an association between cBMI and disease status was present (*n =* 51, patient-level, χ^2^ = 15.0, r = 0.736, AdjP = 0.00182; Fig. 1C; Table S5), suggests that cBMI may act as a confounding variable on further analyses. Notably, this association was not observed when restricting analyzes to HS patients alone (*n =* 33, patient-level, χ^2^ = 4.80, r = 0.520, AdjP = 0.308; Fig. 1D; Table S5). Second, univariate analyses revealed that treatment type (methotrexate and biologicals), age, sample location, abscess lesions, and smoking behavior had minimal impact on phageome composition. Their explanatory power was markedly lower than that of disease severity and cBMI and did not contribute independent effects in the multivariate model (Fig. 1B; Table S4). As such, further analyses will focus on the presence and severity of disease and cBMI as the principal drivers of phageome variation. Given the lack of multivariate effects from longitudinal (i.e., timepoints) or spatial factors (i.e., sample location), the 99 phage-positive samples were pooled per individual, resulting in 51 patient-level profiles for downstream analyzes. Taken together, these findings suggest that together with cBMI, disease presence and severity were key covariates of the phageome composition, marking a diverging viral profile in the progression of HS pathology.

### HS patients exhibit a distinctive skin virome that is highly individualistic and is marked by a reduced healthy core phageome

**Fig 2:**
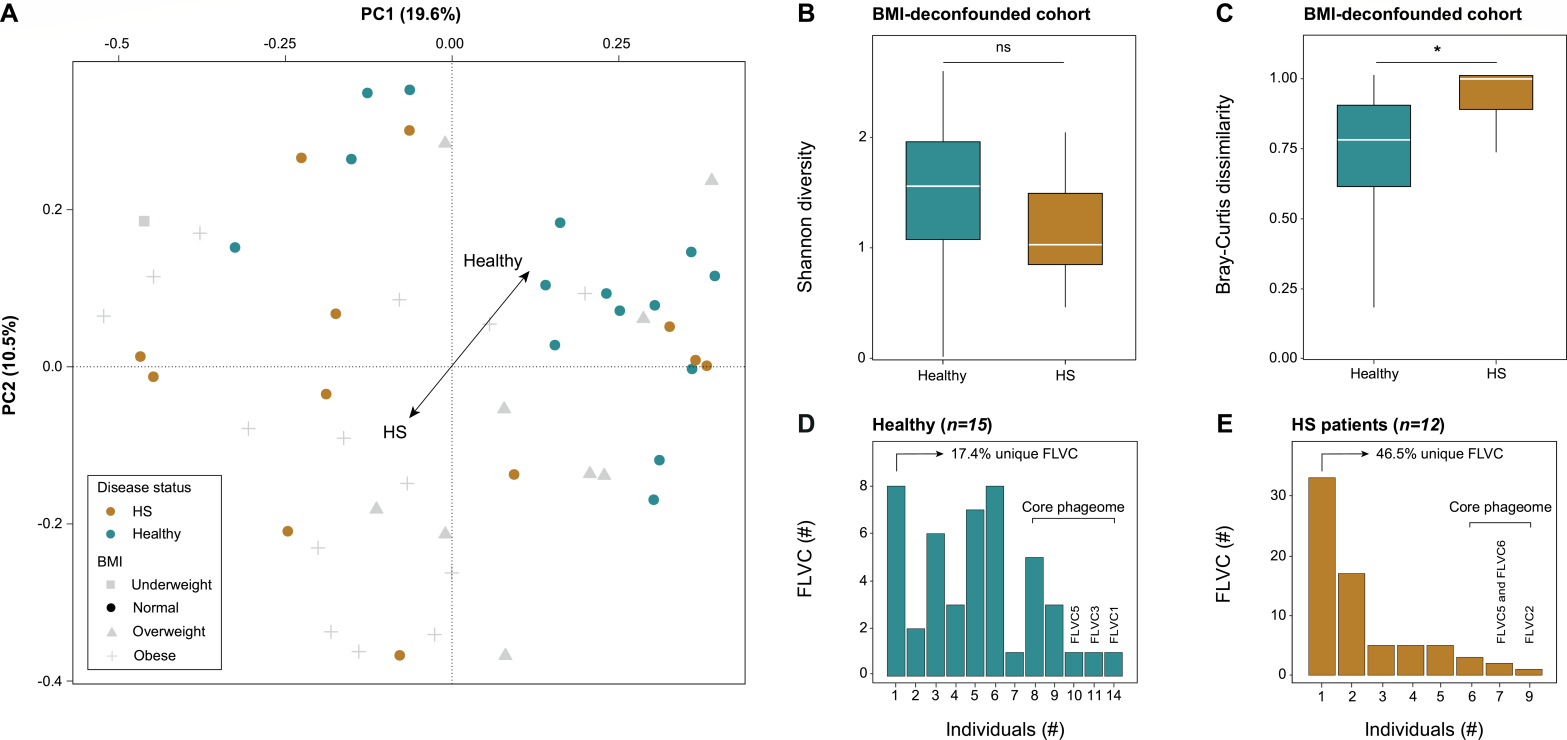
Skin virome diversity and viral sharing in the BMI-deconfounded study cohort. (**A**) A principal coordinate analysis visualizing the inter-individual differences of the skin virome composition (*n =* 51, FLVC level, Bray-Curtis dissimilarity) of the complete cohort (Healthy + HS) colored by disease status and with shapes depicted by categorical BMI. Individuals whose BMI falls outside the normal range (17.5–25 kg/m^2^) are shaded in gray. (**B**) Alpha diversity (Shannon index) boxplot stratified according to disease status in BMI-deconfounded cohort (*n =* 27, FLVC level, BMI = normal, Mann-Whitney U, AdjP ≥ 0.05). (**C**) Beta diversity (Bray-Curtis dissimilarity) boxplot stratified according to disease status in BMI-deconfounded cohort (*n =* 27, FLVC level, BMI = normal, Mann-Whitney U, AdjP = 1.00e-12). (**D**) Barplot showing the absolute and relative number of FLVCs shared within healthy individuals in BMI-deconfounded cohort (*n =* 15*,* BMI = normal). (**E**) Barplot showing the absolute and relative number of FLVCs shared between HS patients in BMI-deconfounded cohort (*n =* 12*,* BMI = normal). The core virome is defined by FLVCs shared between 50% or more of healthy individuals and HS patients (three most shared viruses are listed). Multiple testing adjustment (Benjamini-Hochberg method) was performed, and significant associations (AdjP < 0.05) are represented by an asterisk (*). Abbreviations: hidradenitis suppurativa (HS)/Healthy controls (Healthy), complete cohort (Healthy + HS), family-like viral cluster (FLVC), and non-significant (ns).

Having established the link between the phageome and HS pathology, our focus shifted towards examining how skin phages might differentiate healthy controls from disease. For this purpose, a BMI-deconfounded cohort (only using patients and controls with cBMI = normal) was set up, consisting of 12 HS patients and 15 healthy controls (Fig. 2A). Subsequent diversity analyses did not show a significant difference in alpha-diversity (i.e., Shannon diversity) between HS patients and healthy controls (*n =* 27, Mann-Whitney U, FLVC-level, r = 0.197, AdjP = 0.608; Fig. 2B; Table S6). However, analysis of beta-diversity using Bray-Curtis dissimilarity revealed significantly greater inter-individual heterogeneity in virome composition among HS patients compared with healthy controls (*n =* 27, Mann-Whitney U test comparing distributions of within-group pairwise dissimilarities, FLVC-level, r = 0.552, AdjP = 2.51e-12; Fig. 2C; Table S6). This increased beta-diversity indicated that patients had a far more personalized skin phageome in comparison to healthy controls. To corroborate this finding, we determined the degree of individuality by measuring the proportion of phage FLVC that were unique to a single individual (referred to as “unique FLVC”). Healthy controls had a total of 17.4% unique FLVC, while patients had a total of 46.5% unique FLVC (Fig. 2D and E). To explore viral sharing in greater detail, we focused on phage FLVCs that were detected in 50% or more of individuals within each group independently, that is, either among healthy individuals or among HS patients (Fig. 2D and E). These FLVCs will be referred to as the “core skin phageome” further on. A total of 13 core FLVCs (cFLVCs) were identified in the BMI-deconfounded cohort (Table S7). Among them, four cFLVCs were found in both healthy controls and HS patients, while 7 cFLVCs and 2 cFLVCs were exclusively found in healthy controls and HS patients, respectively (Table S7). In addition, a similar analysis was conducted to investigate cFLVC among different Hurley stages, which showed that cFLVC4 and cFLVC5 were present in all of the patients with severely progressed skin disease (Hurley stage 3; Table S6). Finally, alpha and beta-diversity analyses were also conducted on the non-BMI deconfounded cohort, revealing similar outcomes, underscoring a minimal significance of BMI in the results (Fig. S5; Tables S6 and S7).

**Fig 3:**
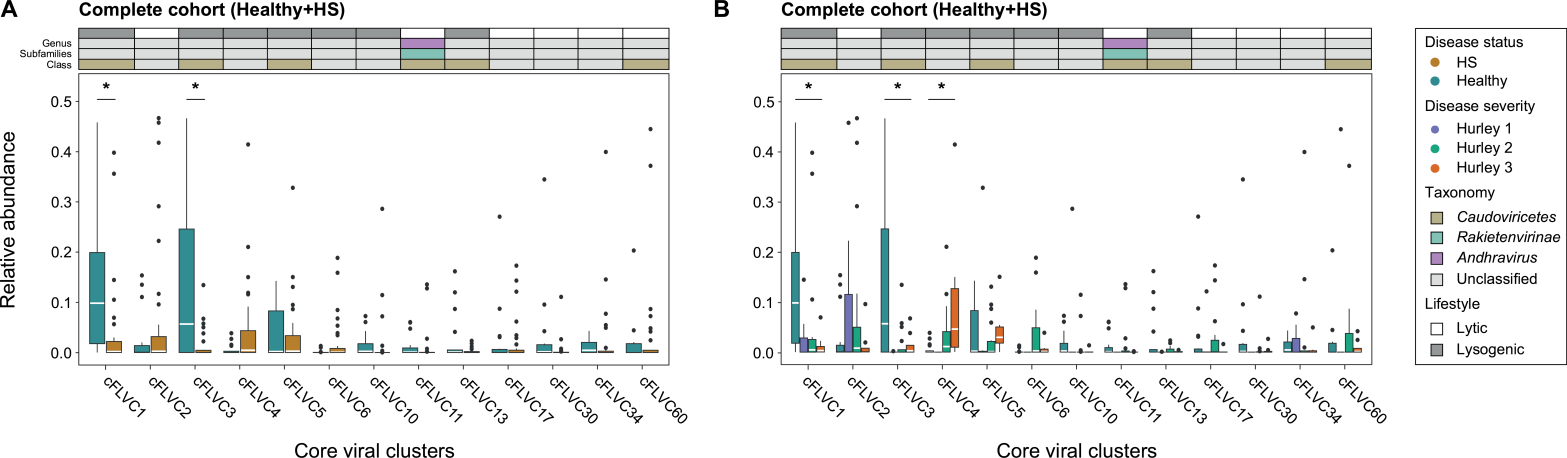
The relative abundance of the cFLVCs in the complete study cohort. (**A**) Relative abundance boxplot of the cFLVCs stratified according to disease status in the complete cohort (*n =* 51, FLVC level, Mann-Whitney U, AdjP < 0.05). The core virome is defined by FLVCs shared between 50% or more of healthy individuals and HS patients. (**B**) Relative abundance boxplot of the cFLVCs stratified according to disease severity in the complete cohort (*n =* 51, FLVC level, Kruskal-Wallis, AdjP < 0.05). Classification (AS ≥ 0.1) and lifestyle of representative high-quality phages of cFLVCs are shown (top) at class, subfamily, and genus taxon. Multiple testing adjustment (Benjamini-Hochberg method) was performed and significant associations (AdjP < 0.05) are represented by an asterisk (*). Abbreviations: alignment score (AS), Hidradenitis suppurativa (HS)/Healthy controls (Healthy), complete cohort (Healthy + HS), family-like viral cluster (FLVC), and non-significant (ns).

Taking these findings into account, we hypothesized that the phage cFLVC could distinguish the skin of healthy controls from that of HS patients and subsequently compared the RA of the 13 previously identified cFLVC between both groups. Healthy controls showed a significantly higher RA of both cFLVC1 and cFLVC3 compared with HS patients (*n =* 51, Mann-Whitney U, FLVC-level, AdjP < 0.05; Fig. 3A; Table S9). In comparison, none of the cFLVCs were found to be significantly higher among HS patients compared with healthy controls (*n =* 51, Mann-Whitney U, FLVC-level, AdjP ≥ 0.05; Fig. 3A; Table S9). Upon classification of these cFLVCs, it was observed that cFLVC1 and cFLVC3 bore no resemblance to phages in RefSeq (Fig. S6). However, cFLVC3 contained a few members showing similarities as high as 95.7% across 50% of their genomes to “uncultured *Caudovirales* phage” genomes in public databases (Table S10), suggesting that they represent a group of poorly described phages within the *Caudoviricetes* class with lysogenic potential. Collectively, these results suggest the existence of a core phageome in healthy individuals, which is depleted in patients with HS.

### Patients with advanced disease are associated with an unidentified phage cluster

Following the inability to identify any cFLVC positively associated with HS, we hypothesized that strong inter-patient variability, combined with the influence of disease progression, may obscure significant associations. Therefore, the RA of the 13 previously identified cFLVCs was compared across different stages of disease severity (Fig. 3B; Tables S11 and S12). Despite the lack of significant differences in RA between the various disease stages for cFLVC1 and cFLVC3 (Kruskal-Wallis test, FLVC-level, AdjP ≥ 0.05), a notably lower RA was identified when compared with healthy controls, consistent with previous findings (Kruskal-Wallis with post-hoc Dunn test, FLVC-level, AdjP <0.05). A significant association between disease severity and cFLVC4 was observed, displaying a gradual rise in RA with the progression of the disease (*n =* 51, Kruskal-Wallis, FLVC-level, r = 0.334, AdjP = 0.00408; Fig. 3B; Table S11). More specifically, the RA of cFLVC4 was significantly higher in advanced disease (i.e., Hurley stage 3, *n =* 26, post-hoc Dunn test, AdjP = 0.00591) but not in mild and moderate disease (i.e., Hurley stage 1 and 2, post-hoc Dunn test, AdjP ≥0.05) compared with healthy controls. Furthermore, patients with moderate and advanced disease showed a significantly higher RA of cFLVC4 compared with mild disease (post-hoc Dunn test, AdjP < 0.05), but were not statistically different from each other (post-hoc Dunn test, AdjP ≥ 0.05). A more detailed analysis of phage genomes in cFLVC4 showed no similarities to phage genomes in RefSeq (Fig. S6) or other public databases (Table S10), suggesting that it corresponds to a previously unidentified phage cluster. Genomic features indicate the potential for lysogenic conversion, though direct experimental evidence is needed to confirm this (Table S10). Taken together, these results point toward the existence of a core phage FLVC that gains more ground in the later stages of HS.

### The healthy core phageome is predicted to infect *Corynebacterium*, and *Staphylococcus* and is marked by a high presence of prophage-mediated bacterial defense systems

**Fig 4:**
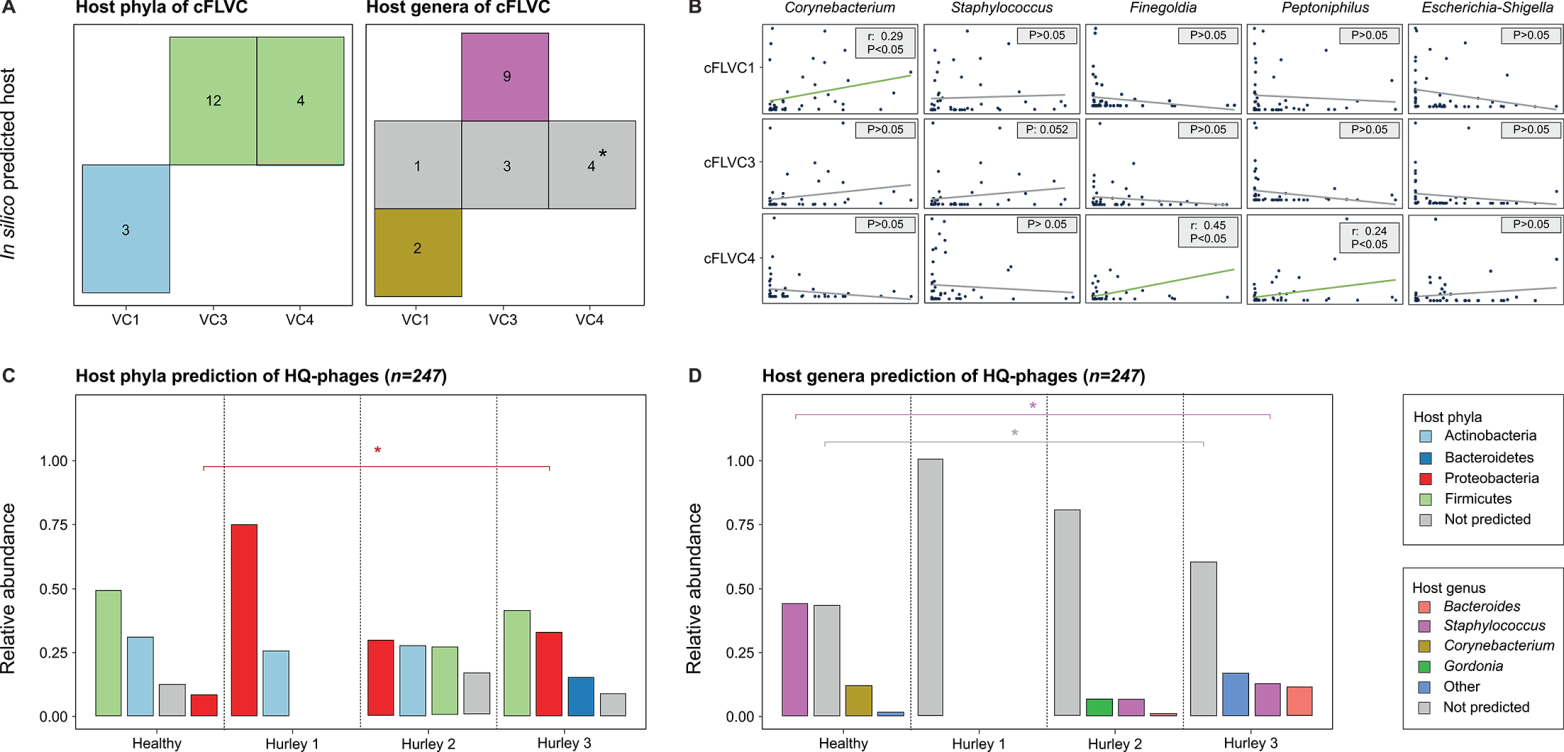
Host prediction of the skin virome. (**A**) Tile plot of *in silico* predicted bacterial host phyla (left) and genera (right) of high-quality phage representative sequences of the core viral clusters significantly associated with disease status and disease severity. The number of representatives of each FLVC is depicted in each tile and is near-complete (>50% complete) phage sequences. The asterisk denotes that a comparison of phage-incorporated bacterial proteins of the representatives of FLVC4 to protein databases could provide insights into the potential host (i.e., *Finegoldia* and *Peptoniphilus*; Table S12). (**B**) Comparison of the relative abundance of core viruses to the relative abundance of predicted bacterial genera, as determined by 16S bacterial data (38). The Escherichia-Shigella group (most abundant genera) was not predicted by former analysis and served as a negative control. (**C**) Compositional barplot of *in silico* predicted bacterial host phyla of high-quality phages stratified according to disease severity (*N =* 43, Kruskal-Wallis, AdjP < 0.05). (**D**) Compositional barplot of *in silico* predicted bacterial host genera of high-quality phages stratified according to disease severity (*N =* 43, Kruskal-Wallis, AdjP < 0.05). *In silico* host predictions are determined with random forest assignment of hosts (RaFAH). Multiple testing adjustment (Benjamini-Hochberg method) was performed. Abbreviations: family-like viral cluster (FLVC).

Having identified the phages associated with a healthy skin, our next goal was to investigate how these phages might influence the skin microbiome. To do so, we used an *in silico* host prediction tool (i.e., RaFAH) to forecast the likely bacterial host. Based on this, it was predicted that cFLVC1 phages target Actinobacteria, whereas cFLVC3 phages infect Firmicutes (Fig. 4A; Table S10) (43). To obtain a more refined taxonomical resolution, *in silico* host predictions were repeated to estimate the bacterial genus. The findings indicated that cFLVC1 phages primarily target *Corynebacterium* species, while cFLVC3 phages predominantly infect *Staphylococcus* species (Fig. 4A; Table S10). Furthermore, 16S bacterial data obtained from a parallel study was used to confirm the association between the healthy core phages and their respective bacterial host (Fig. 4B; Table S13) (38). A notable positive correlation was found between the RA of cFLVC1 and the RA of *Corynebacterium* (*n =* 51, r = 0.29, AdjP = 0.039; Fig. 4B; Table S13), providing further evidence of a connection between these phages and their bacterial hosts. Moreover, no significant correlation was detected between the RA of cFLVC3 and the RA of *Staphylococcus* (*n =* 51, AdjP = 0.052; Fig. 4B; Table S13). This lack of a significant correlation may be due to the diverse nature of *Staphylococcus* on the skin (44), which include both symbiotic (i.e., *Staphylococcus epidermidis*) and pathogenic species (i.e., *Staphylococcus aureus*), that could be associated with phages other than cFLVC3. However, this could not be confirmed due to the limited reliability of species-level host predictions using *in silico* methodologies. In addition, the bacterial genus *Escherichia-Shigella*, serving as a negative control for the correlation analysis, was found to have no significant association with either cFLVC1 or cFLVC3 (*n = 51*, AdjP <0.05; Fig. 4B; Table S13).

**Fig 5:**
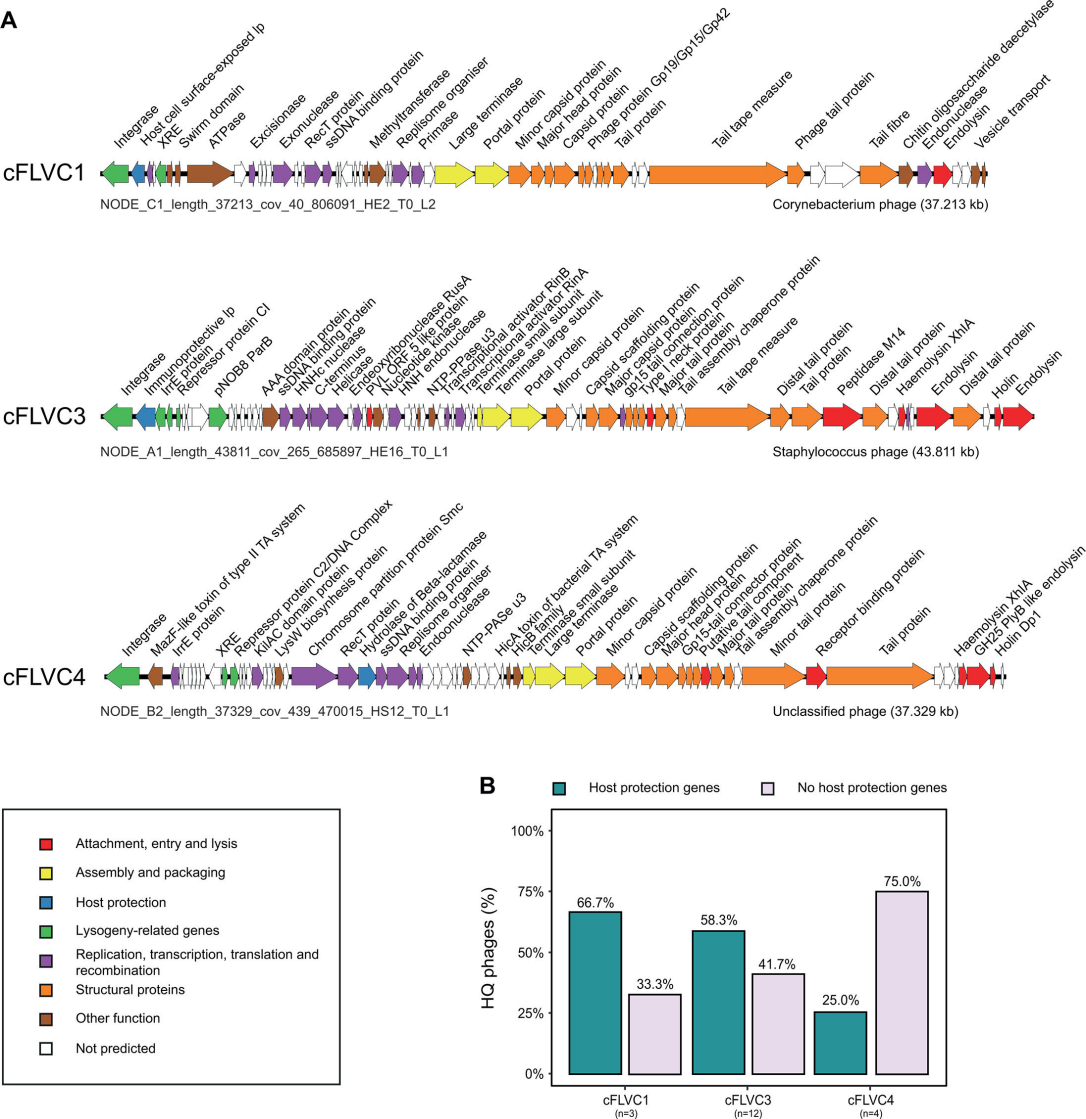
Genome maps of representative high-quality phages for each cFLVC. (**A**) Genome maps of representative high-quality (near-complete) phages of FLVC1, FLVC3, and FLVC4 as obtained by Cenote-Taker2 (46), with arrows representing predicted ORFs. The genomes of FLVC1 (NODE_C1_length_37213_cov_40_806091_HE2_T0_L2) and FLVC3 (NODE_A1_length_43811_cov_265_685897_HE16_T0_L1) were determined to be lysogenic *Corynebacterium* and *Staphylococcus* phages, respectively. The representative of FLVC4 (NODE_B2_length_37329_cov_439_470015_HS12_T0_L1) is determined to be a lysogenic phage with unpredicted host genus using RaFAH (43). Phage genomes are linearized to improve visualization. (**B**) Percentage of high-quality phages containing genes advantageous to the bacterial host (by increasing host fitness) stratified according to each cFLVC. Abbreviations: family-like viral cluster (FLVC) and high-quality phages (HQ phages).

To complete our analysis of the healthy core phageome, the representative phage genomes of cFLVC1 and cFLVC3 were functionally annotated, as shown in Fig. 5A. These genome maps revealed the predicted presence of proteins that may enhance fitness of the bacterial host and were found within the majority of individual phage genomes (i.e., cFLVC1 = 66.7%, cFLVC3 = 58.3%; Fig. 5B; Table S10). Among these predicted proteins were lipoproteins, such as “host cell surface-exposed lipoprotein” and “Siphovirus gp157 protein,” located within the lysogeny module of the phage genomes. These proteins were previously recognized for their involvement in “superinfection exclusion,” a mechanism that safeguards the bacterial cell by preventing secondary viral infections (45). Collectively, the results suggest that phages present on the skin of healthy individuals could integrate their genetic material into the genomes of *Corynebacterium* and *Staphylococcus*, potentially enhancing bacterial fitness through mechanisms such as superinfection exclusion; however, this hypothesis remains to be experimentally validated.

Finally, we conducted an *in silico* host prediction on the complete phageome, expanding our analysis beyond individual viral clusters (Fig. 4C and D). The results revealed that, at the phylum taxonomy level, there is a significantly higher RA of proteobacteria-infecting phages in HS patients compared with healthy controls (Kruskal-Wallis with post-hoc Dunn test, AdjP < 0.05, Fig. 4C; Table S14). Analysis of the genus level not only indicated a lower RA of *Staphylococcus*-infecting phages, but also a higher RA of phages without an assigned host in HS patients compared with healthy controls (Kruskal-Wallis with post-hoc Dunn test, AdjP < 0.05; Fig. 4D; Table S14).

### Disease-associated core phages potentially infect *Finegoldia* and *Peptoniphilus* and revealed the presence of antibiotic resistance genes

As mentioned previously, disease-associated core phages (i.e., cFLVC4) gradually gained more ground in later stages of HS, eventually establishing a presence in all patients with advanced disease. To further understand the impact of these phages on the skin microbiome of patients, we conducted *in silico* host prediction analyses. The analysis revealed that cFLVC4 phages showed a preference for infecting Firmicutes; however, accurate predictions at the bacterial genus could not be made (Fig. 4A; Table S10). As an alternative strategy to gain insights into their potential host, phage-incorporated bacterial proteins found within the phage genomes were explored, such as Haemolysin XhlA or Chromosome partition protein Smc (Table S10). The underlying hypothesis was that these proteins, acquired from bacteria during past infections, could offer clues about the phage’s current bacterial host. Comparison of such proteins with bacterial databases suggested that cFLVC4 phages likely target *Peptoniphilus* (best blastp hit: AAI% = 99.0%; coverage = 96.3%) and *Finegoldia* (best blastp hit: AAI% = 95.3%; coverage = 100%), both known pathogens associated with HS (47). In addition, the RA of both bacterial genera showed a positive and significant correlation with the RA of cFLVC4 phages (*n = 51*, r > 0.30, AdjP < 0.05; Fig. 4B; Table S13), adding further evidence to support the relationship between these phages and their host. However, it is important to note that the detected phage-incorporated bacterial proteins may represent remnants of past infections and may not necessarily reflect the phage’s current host.

Finally, after carefully analyzing the genome maps of the disease-associated core phages, we incidentally observed the presence of antibiotic resistance genes, although only in a minority of representative phage genomes (i.e., hydrolase of beta-lactamase; cFLVC4 = 25.0%, Fig. 5B; Table S10). These genes have the potential to enhance bacterial fitness by conferring resistance to beta-lactam antibiotics. Taken together, the collective evidence suggests that phages present in advanced disease may integrate their genetic material into the genome of *Peptoniphilus* and *Finegoldia*. This integration could potentially enhance bacterial fitness by providing antibiotic resistance genes, though further functional studies are necessary to confirm this role and its clinically relevant in HS pathology.

## DISCUSSION

To the best of our knowledge, this research represents the first investigation of the skin virome in patients diagnosed with hidradenitis suppurativa. Here, we found a small yet diverse eukaryotic virome with anelloviruses confined to disease. Earlier studies have already indicated that anelloviruses were rarely found on the skin of healthy individuals (48), suggesting a potential association with the inflammatory component (i.e., immune cells) present in patients. However, since the host cells and biological functions of anelloviruses in the human body remain largely unknown, this interpretation should be considered with caution. In line with our data, eukaryotic viruses were detected at low abundance and did not vary consistently between healthy and diseased individuals. Therefore, they are unlikely to play a meaningful role in HS pathology. By contrast, when we shifted our attention to the phageome, we found a significant association with HS pathology. This association emerged after employing a viral clustering approach, which allowed us to obtain a more comprehensive representation of the skin phageome. While a majority of the phages could be recognized, only a minority could be classified, indicating the presence of previously uncharacterized phages in our data set.

When considering the relationship between the phageome and HS pathology, it was evident that the phageome exhibited a robust association with both the presence and severity of HS. This indicated that the skin phageome may possess the ability to differentiate, to a certain extent, between healthy individuals and HS patients. To further explore the mechanism by which the phageome achieved this differentiation, we examined various diversity indices. This indicated that patients had a significantly more personalized phageome compared to controls, indicating a reduced level of phage sharing among patients. To gain a deeper understanding of phage sharing, the concept of “core viral clusters” was introduced, which comprised phage clusters that were present in 50% or more of the individuals. Using this concept, two core phage clusters, namely cFLVC1 and cFLVC3, both belonging to the *Caudoviricetes* class, were found to be highly abundant on the skin of healthy individuals but not of HS patients. These phages were identified as lysogenic and capable of infecting *Corynebacterium* and *Staphylococcus*, which comprised known skin commensals. Furthermore, they harbored genes involved in superinfection exclusion, a mechanism that may confer bacterial resistance to a secondary infection of closely related phages. Thus, it was hypothesized that cFLVC1 and cFLVC3 may enhance the fitness of *Corynebacterium* and *Staphylococcus*, thereby stabilizing the microbiota and promoting skin symbiosis. Interestingly, other studies have suggested that genes for superinfection exclusion may be constitutively transcribed during the prophage status, highlighting the intricate role of the phageome within the skin microbiome (45). To conclude our study, we examined different stages of the disease and identified a previously unidentified core phage cluster, namely cFLVC4, that was more prevalent in advanced stages of the disease. This core phage cluster, which was discovered in all patients with late-stage disease (i.e., Hurley stage 3), exhibited a lysogenic nature and likely has the ability to infect *Peptoniphilus* and *Finegoldia*, two known pathogens associated with HS (47). Remarkably, the phages revealed the presence of antibiotic resistance genes, potentially complicating patient treatment and promoting skin dysbiosis. We speculate that this observation is linked to the higher use of antibiotics in late stage (80%) compared to mild or moderate disease (40% and 31.2%; Table S1). It is important to note that our study had a relatively limited sample size, which may affect the generalizability of these findings. Larger cohorts are needed to validate the observed associations, confirm the identified core phage clusters, and further elucidate the role of the phageome in HS pathogenesis.

To summarize, our study suggests a relationship between the skin virome and HS pathology. We identified distinct groups of phages associated with either healthy controls or HS patients, which could promote bacterial fitness. In healthy individuals, these phages could contribute to the stability of the skin microbiota and foster symbiosis through mechanisms like superinfection exclusion. However, in HS patients, the presence of phages could potentially contribute to skin dysbiosis by providing antibiotic resistance genes and complicating treatment, underscoring the clinical significance of the virome in HS pathology.

## Data Availability

Table S1 contains the clinical metadata. The raw sequence data have been deposited to the NCBI Sequence Read Archive with BioProject accession number PRJNA961962. The high-quality phage sequences (representatives of cFLVC1, cFLVC3, and cFLVC4) were deposited to GenBank under the following accession numbers: OQ890309–OQ890326. The ViPER (Virome Paired-End Reads) pipeline was used for bioinformatic processing of the raw paired -end reads and is publicly available at GitHub (https://github.com/Matthijnssenslab/ViPER). All the data to reproduce virome analysis are available at https://github.com/Matthijnssenslab/IBDVirome/tree/main/IBDHS.

## SUPPLEMENTAL MATERIAL

Supplemental Figures msystems.01290-25-s0001.docx Figures S1 to S6.

Supplemental Text msystems.01290-25-s0002.docx Extended methods.

Supplemental Tables msystems.01290-25-s0003.xlsx Tables S1 to S14.

ASM does not own the copyrights to Supplemental Material that may be linked to, or accessed through, an article. The authors have granted ASM a non-exclusive, world-wide license to publish the Supplemental Material files. Please contact the corresponding author directly for reuse.
