# Supplementary material for: Hidradenitis suppurativa patients exhibit a distinctive and highly individualized skin virome
Source: mSystems. 2025 Dec 15;11(1):e01290-25. doi: 10.1128/msystems.01290-25 (PMC12817917; doi:10.1128/msystems.01290-25)
Supplement: Supplemental Figures — Figures S1 to S6. [file msystems.01290-25-s0001.docx]

**EXTENDED DATA FIGURES**

**Figure 1: Bioinformatic analysis of the skin virome.** An overview of the bioinformatics (dry lab) analysis, also known as drylab analysis, of the skin virome. First, raw reads undergo trimming and decontamination to acquire high-quality reads, referred to as “QC-Reads”. QC-reads are then *de novo* assembled three times, using subsets of reads, resulting in long contiguous sequences through a process called “triple assembly”. Next, contigs are clustered to obtain a non-redundant contig set. Viruses are identified using specific methods outlined in the methods sections, classified and abundances are calculated. Additionally, the functional characteristics of the identified viruses and their hosts are determined through further analysis. Abbreviations: Input/Output (I/O), Contiguous sequences (contigs), non-redundant (NR) and viral cluster (FLVC).

**Figure 2: The quality control steps implemented to obtain the viral metagenome of the complete study cohort. A,** The number of reads obtained during each quality control step for the HS dataset is presented, along with a comparison of identifying only high-quality phages versus using the viral cluster approach to identify phages (raw reads=4.15 billion). Viral reads (4.27%-7.08% of raw reads) represent quality-controlled reads mapped to viruses identified with (number of viral reads=294 million, %viral reads=66.9%) or without (number of viral reads=177 million, %viral reads=40.2%) the viral cluster approach. **B,** Pie chart depicting the distribution of viral reads, mapping to either prokaryotic or eukaryotic viruses, stratified based on the viral identification approaches, as described before. Abbreviations: Hidradenititis suppurativa (HS), Complete cohort (Healthy+HS) and family like viral cluster (FLVC).

**Figure 3: The classified and unclassified phageome in the complete study cohort. A,** The percentage of classified and unclassified non-redundant phage contigs in the high-quality phage (*n=247* NR-contigs) and FLVCs (*n=2134* NR-contigs) dataset. **B,** The percentage of classified and unclassified non-redundant phage contigs in the high-quality phage dataset on class taxon (25.5% classified and 74.5% unclassified). **C,** The percentage of classified and unclassified non-redundant phage contigs in the viral cluster phage datasets on class taxon (41.2% classified and 59.8% unclassified). The classification of phages was performed by homology-based (AS ≥ 0.1) and marker gene classification (see Methods). Abbreviations: Alignment score (AS), Complete cohort (Healthy+HS), high-quality contigs (HQ-contigs) and non-redundant contigs (NR-contigs).

**Figure 4: The eukaryotic viral composition in the complete study cohort. A,** A relative abundance heatmap depicting the eukaryotic virome in HS patients and healthy controls (family level, *n=38*, eukaryotic viral presence=66.7%). The eukaryotic viral families have been categorized into groups based on their hosts, which are either known (plant/fungal and animal viruses) or unknown (small circular viruses). The classification of eukaryotic viruses was performed by homology-based classification (AS ≥ 0.1). **B,** Relative abundance boxplots of the most prevalent viral families (*Alphaflexiviridae*, *Anelloviridae*, *Totiviridae* and *Virgaviridae*) stratified according to disease status in complete cohort (Mann-Whitney U, AdjP≥0.05). Adjustment for multiple testing was performed using the Benjamini-Hochberg method. Abbreviations: Alignment score (AS), Complete cohort (Healthy+HS), Relative abundance (RA) and not-significant (ns).

**Figure 5: Skin virome diversity and viral sharing in the complete study cohort. A,** A principal Coordinate Analysis visualizing the inter-individual differences of the skin virome composition (*n=51*, FLVC level, Bray-Curtis dissimilarity) of the complete cohort (Healthy+HS) coloured by disease status and with shapes depicted by categorical BMI. **B,** Alpha diversity (Shannon index) boxplot stratified according to disease status in complete cohort (*n=51*, FLVC level, Mann-Whitney U, AdjP≥0.05). **C,** Beta diversity (Bray-Curtis dissimilarity) boxplot stratified according to disease status in complete cohort (*n=51*, FLVC level, BMI=normal, Mann-Whitney U, AdjP< 2.2e-16). **D,** Barplot showing the absolute and relative number of FLVC shared between healthy individuals (*n=18*). **E,** Barplot showing the absolute and relative number of FLVC shared within HS patients (*n=33*). The core virome is defined by FLVC shared between 50% or more of healthy individuals or HS patients (most shared viruses are listed). Multiple testing adjustment (Benjamini-Hochberg method) was performed and significant associations (AdjP<0.05) are represented by an asterisk (*). Abbreviations: Hidradenititis suppurativa (HS)/Healthy controls (Healthy), Complete cohort (Healthy+HS), viral cluster (FLVC) and non-significant (ns).


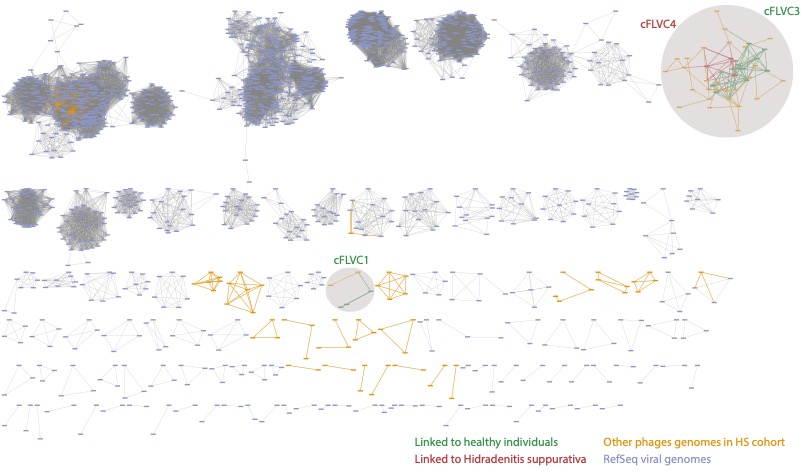
 **Figure 6: vConTACT2 clustering method for grouping phages at a genus-like level.** Illustration of viral clusters found after combining the high-quality phages (*n=247*) with the current (April 14, 2023) RefSeq viral database (*n=4406*). The nodes and edges in the network of the three viral clusters (FLVC1, FLVC3 and FLVC4) that are capable of differentiating between disease status and disease severity are colored (green/red) and highlighted with a gray circle. There was not a single connection found between the aforementioned viral clusters and the genomes present in the current RefSeq viral database (i.e., no single RefSeq genome within gray encircled area). There was also not a single connection found between the high-quality phages within FLVC3 and FLVC4 as they produced their own vConTACT2 clusters (Supplementary Table 12). Abbreviations: family like Viral cluster (FLVC).
